# Classification of Insomnia Using the Traditional Chinese Medicine System: A Systematic Review

**DOI:** 10.1155/2012/735078

**Published:** 2012-07-30

**Authors:** Maggie Man-Ki Poon, Ka-Fai Chung, Wing-Fai Yeung, Verdi Hon-Kin Yau, Shi-Ping Zhang

**Affiliations:** ^1^Department of Psychiatry, The University of Hong Kong, Pokfulam Road, Hong Kong; ^2^School of Chinese Medicine, Hong Kong Baptist University, Hong Kong

## Abstract

A systematic review was conducted to examine traditional Chinese medicine (TCM) patterns commonly diagnosed in subjects with insomnia and clinical features associated with the TCM patterns, and an insomnia symptom checklist for TCM diagnostic purpose was developed based on the review. Two independent researchers searched the China Academic Journals Full-Text Database and 10 English databases. A total of 103 studies and 9499 subjects were analyzed. There was a wide variation in terminology relating to symptomatology and TCM pattern. We identified 69 patterns, with the top 3 patterns (i.e., *deficiency of both the heart and spleen, hyperactivity of fire due to yin deficiency, and liver-qi stagnation transforming into fire*) and the top 10 patterns covering 51.8% and 77.4% of the 9499 subjects, respectively. There were 19 sleep-related, 92 non-sleep-related, 14 tongue, and 7 pulse features included as diagnostic criteria of the top 10 TCM patterns for insomnia. Excessive dreaming, dizziness, red tongue, and fine pulse were the most common sleep-related, non-sleep-related, tongue, and pulse features. Overlapping symptomatology between the TCM patterns was present. A standardized symptom checklist consisted of 92 items, including 13 sleep-related, 61 non-sleep-related, 11 tongue, and 7 pulse items, holds promise as a diagnostic tool and merits further validation.

## 1. Introduction

Insomnia is the most common sleep complaint, with approximately 9–15% of the general population worldwide suffering from insomnia symptoms accompanied by daytime consequences [1]. Insomnia is associated with psychological distress, impaired daily functioning, and an increased risk of medical and psychiatric morbidity and mortality [2]. Although effective pharmacologic and psychological treatments for insomnia are available, their uses are limited due to concerns regarding adverse effects and feasibility in everyday clinical settings [3, 4]. Faced with the limitations of the currently available treatments, complementary and alternative medicine (CAM) has been sought to treat insomnia. A national survey in the United States showed that 4.5% of adults reported using some form of CAM for insomnia in the past year [5]. Traditional Chinese medicine (TCM), a form of CAM, is one of the oldest medical systems in the world. A population-based study in Australia showed that around 20% of adults used at least one form of TCM treatments in the past year [6]. A study in Taiwan showed that 28% of valid beneficiaries of the national health insurance filed claims for TCM treatment during the year 2002 [7].

The recognition of insomnia as a major health problem can be traced back to more than 2000 years ago in ancient Chinese medical texts [8, 9]. Based on the patient's symptoms and signs, TCM practitioners describe the patterns of bodily disharmony in terms of eight major parameters: *yin* and *yang*, *external* and *internal*, *hot* and *cold*, and *excess* and *deficiency*. Additional systems, such as *qi*, *blood* and *body-fluid* differentiation, and *zang fu* (organ) differentiation are also used [10]. The TCM patterns describe differences in etiology and pathogenesis of diseases and emphasize variation in individuals' body constitution. Although most of the TCM concepts have yet been proven by scientific method, the TCM diagnostic system continues to be practiced nowadays. Treatment principles and specific herbal formula or acupoints are derived according to the TCM pattern. Nevertheless, the key shortcomings of the TCM diagnostic process are the lack of standardization in terminology and disagreement on pattern differentiation among Chinese medicine practitioners [11–14].

To the best of our knowledge, there has been no systematic assessment of the reliability and validity of the TCM pattern differentiation for insomnia. Although the publication of standard TCM textbooks in China can be seen as an attempt to minimize disagreement among practitioners, the recognition and acceptance of the textbooks among TCM practitioners are uncertain. Given the frequent occurrence of insomnia among patients presenting to TCM practitioners, it is important to use standardized terminology and criteria for TCM diagnosis. As a first step of the standardization, we conducted a systematic paper of TCM patterns commonly diagnosed in subjects with insomnia and gathered information on the clinical features of the TCM patterns. Based on our review, we constructed an insomnia symptom checklist which could be used as a diagnostic tool for future research and clinical purposes.

## 2. Material and Methods

### 2.1. Search Strategy

We searched the Cochrane Central Register of Controlled Trials (CENTRAL), MEDLINE, EMBASE, PsycINFO, PUBMED, Dissertation Abstracts International, Cumulative Index to Nursing and Allied Health Literature (CINAHL), Allied and Complementary Medicine Database (AMED), National Center for Complementary and Alternative Medicine, National Institute of Health Clinical Trials Database, China Academic Journals Full-Text Database from inception to November 2008 using the grouped terms “Chinese medicine or TCM or acupunc* or acupress* or electroacupunc* or meridian* or acupoint* or tuina*” and “sleep* or insomnia* or wakeful* or sleepless* or somnambul*” and the China Academic Journals Full-Text Database using equivalent Chinese terms. The reference lists of the retrieved papers were further searched for relevant articles.

### 2.2. Selection Criteria

We included studies that described TCM patterns of subjects with a chief complaint of insomnia. In order to obtain a full coverage of the topic, we did not set any specification for sampling procedure, treatment method, outcome measure, and study quality. Aiming to derive a general picture of TCM pattern utilization, studies were excluded if they (1) had less than 30 subjects; (2) examined males or females only; (3) focused on individuals aged below 18 or above 70 years; (4) focused on a specific medical and psychiatric condition, a particular life transition period, or a specific TCM pattern; (5) had no statistical information regarding TCM pattern; (6) were duplicated publications. The authors (MKP and HKY) searched the databases and selected the relevant publications independently. Full papers of the relevant publications were obtained and reviewed in detail against the inclusion and exclusion criteria. Any disagreement about the eligibility of study was resolved by thorough discussion.

### 2.3. Data Extraction Process

For each study, the following variables were extracted: study design, sample size, mode of recruitment, sampling and diagnostic procedure, inclusion and exclusion criteria, and participants' characteristics including age, gender, and duration of insomnia. Information regarding the TCM pattern including symptoms and signs of each TCM pattern was obtained. All Chinese to English translations were deduced primarily from the *World Health Organization *(*WHO) International Standard Terminologies on Traditional Medicine in the Western Pacific Region *[15] and additionally from *the Traditional Chinese Internal Medicine *[16], a widely used English-language TCM textbook in China.

### 2.4. Construction of an Insomnia Symptom Checklist for TCM Diagnostic Purpose

The symptom checklist included clinical features of the 10 most common TCM patterns associated with insomnia. The top 10 TCM patterns were chosen because they covered roughly 80% of subjects with insomnia (Table 1). If more TCM patterns were covered, the symptom checklist would be too lengthy. Symptoms included in the checklist needed to have mentioned as clinical features of the TCM patterns in at least 10% of the reviewed studies; thus, both common and less common features would be listed. In addition, we reviewed several standard TCM textbooks for colleges and universities, including the editions of *Traditional Chinese Internal Medicine* published in 1985 [17], 1997 [18], 2003 [19], and 2007 [16] for symptoms that were not described in the reviewed studies.

## 3. Results

### 3.1. General Description of the Reviewed Studies

The search yielded 4795 potentially relevant citations, of which 3036 citations were excluded for reasons of irrelevance or duplication. A total of 1759 articles that were related to insomnia and TCM were retrieved for further review. Three hundred thirty-six articles were discussion papers, 95 were restricted to subjects aged below 18 or above 70 years, 33 focused on either males or females, 73 were limited to specific medical and psychiatric conditions, 19 focused on a particular life transition period, 145 were studying a specific TCM pattern, 264 had less than 30 subjects, 364 did not have information on TCM pattern, 310 had no statistical information regarding the frequency of individual TCM pattern, 16 were written neither in Chinese nor English, and one could not be retrieved in full text. These 1656 studies were excluded and the remaining 103 studies were included in this paper. Full details of the excluded studies are available from the authors.

The sample size of the 103 studies ranged from 30 to 856. TCM diagnosis was available in 9499 subjects. Based on the sex distribution, mean age, and number of subjects reported in each study, about 56.5% of the total sample were female and the subjects' mean age were 44.0 years. All included studies were conducted in China, and 5 (4.9%) were published in English-language journals. The criteria used for diagnosis of insomnia varied between studies. Twelve of the 103 studies (11.7%) were based on the *Criteria of Diagnosis and Therapeutic Effect of Diseases and Syndromes in Traditional Chinese Medicine *[20], 11 (10.7%) studies used the *Chinese Classification of Mental Disorder *[21], nine (8.7%) used the *Clinical Research Guidelines of New Chinese Herbal Medicine *[22], and one (1.0%) used the* WHO* diagnostic criteria [23]. Thirty-six (35.0%) studies based on TCM textbook or other criteria, and 34 (33.0%) did not report the diagnostic criteria used.

### 3.2. TCM Pattern Differentiation for Insomnia

Seventy-four different TCM patterns were reported in the 103 included studies. Similar patterns were grouped together. Thus, *heart-gall bladder deficiency and timidity* (*心膽虛怯*) was grouped under *heart deficiency with timidity* (*心虛膽怯*);* heart and spleen deficiency* (*心脾虧損*) was considered as *heficiency of both the heart and spleen* (*心* *脾* *兩虛*); and *stomach lost harmony* (*胃腑失和*) was grouped under* stomach disharmony* (*胃腑不和*); *stomach qi lost harmony* (*胃氣失和*) was grouped under *stomach qi disharmony* (*胃氣不和*); *phlegm-fire hindering the heart* (*痰火撓心*) was considered as *phlegm-fire harassing the heart* (*痰火擾心*). After grouping similar patterns, a total of 69 TCM patterns had been used for classification of insomnia. The most commonly presented pattern was *deficiency of both the heart and spleen *(*N* = 2378, 25.0% of the 9499 subjects), followed by *hyperactivity of fire due to yin deficiency*, *liver-qi stagnation transforming into fire*,* heart-kidney noninteraction*, *qi deficiency of the heart and gallbladder*, *internal disturbance of phlegm-heat*, *liver fire flaming upward*, *heart deficiency with timidity*, *stomach disharmony*, and *stomach qi disharmony*. The top 10 TCM patterns accounted for 77.4% of the 9499 subjects (Table 1).

### 3.3. Terms Relating to Sleep-Related, Non-Sleep-Related, Tongue, and Pulse Features

Thirty-seven of the included studies provided clinical features of individual TCM patterns. We examined sleep-related, non-sleep-related, tongue, and pulse features of the 10 most commonly presented TCM patterns. A total of 52 Chinese terminologies relating to sleep-related symptoms were mentioned, but many had similar meaning. For example, eight different Chinese terminologies were used to describe difficulty falling asleep and four different Chinese terms describing insomnia. After grouping similar terms, there were 19 different sleep-related symptoms. In the order of frequency, the terms included excessive dreaming, insomnia, difficulty staying asleep, difficulty falling asleep, insomnia with vexation, restless sleep, frequent awakening with a start, half asleep, sleeping late at night, nonrefreshing sleep, early-morning awakening, shallow sleep, daytime sleepiness, easy awakening from sleep with difficulty getting back to sleep, inability to sleep for the whole night, difficulty falling asleep alone, difficulty falling asleep at night, nightmare, and difficulty falling asleep with vexation.

There were 169 Chinese terminologies relating to non-sleep-related symptoms of the 10 most commonly presented TCM patterns for insomnia. After grouping similar Chinese terms, we found 92 non-sleep-related symptoms that were described in the top 10 TCM patterns for insomnia. The more frequently mentioned non-sleep-related symptoms, in the order of frequency, included dizziness, palpitation, vexation, poor memory, dry mouth, tinnitus, bitter taste, lassitude, feverish sensations in the palms, soles, and chest, fatigue, backache, timidity, reduction in luster complexion, irritability, poor appetite, constipation, oppression in the chest, reddish eyes, stuffiness in the chest and stomach, headache, tasteless, yellow urine, and sore knees. Depressed mood and weight loss were only mentioned in one article.

There were 19 Chinese terms relating to tongue features in subjects with insomnia; after grouping similar terms, it was reduced to 14. The tongue features, in the order of frequency, were red tongue, pale tongue, thin coating, yellow coating, slimy coating, scanty coating, and white coating. There were seven pulse features in the TCM classification system related to insomnia complaints. Fine pulse was the most commonly mentioned in patients with insomnia, followed by rapid pulse, string-like pulse, weak pulse, and slippery pulse.

### 3.4. Comparing the 10 Most Commonly Presented TCM Patterns for Insomnia

Based on our paper, we found that most sleep-related symptoms appeared in more than one TCM pattern (Table 2). For example, excessive dreaming and difficulty falling asleep were found in seven of the 10 most commonly presented TCM patterns, while difficulty staying asleep was present in five of the top 10 patterns.

We found that dizziness, vexation, palpitation, tinnitus, and bitter taste were non-sleep-related symptoms that occurred in at least four of the top 10 TCM patterns (Table 2). Dizziness was included as a non-sleep-related symptom in *deficiency of both the heart and spleen*, *hyperactivity of fire due to yin deficiency*, *heart-kidney noninteraction*, and *stomach disharmony*. Vexation was present in all excess patterns except *stomach qi disharmony* and could be found in three deficiency patterns *hyperactivity of fire due to yin deficiency*, *qi deficiency of the heart and gallbladder*, and *heart-kidney noninteraction*. Palpitation was described in all of the deficiency patterns and *liver fire flaming upward*. Tinnitus was present in three excess patterns *liver-qi stagnation transforming into fire*, *liver fire flaming upward*, and *stomach disharmony* and two deficiency patterns *hyperactivity of fire due to yin deficiency* and* heart-kidney noninteraction*. Bitter taste was found in three excess patterns and one deficiency pattern.

The tongue feature which commonly occurred in excess patterns was red tongue (Table 2). For *liver-qi stagnation transforming into fire,* there was an addition of yellow coating, and for *internal disturbance of phlegm-heat*, there was an addition of yellow and slimy coating. However, red tongue could also occur in two deficiency patterns, *hyperactivity of fire due to yin deficiency* and *heart-kidney noninteraction*. Pale tongue was present in all deficiency patterns except *hyperactivity of fire due to yin deficiency;* for *deficiency of both the heart and spleen*, there was an additional thin coating.

The pulse feature which commonly occurred in excess TCM patterns was rapid pulse; for deficiency patterns, it was fine pulse. However, rapid pulse was also found in the two deficiency patterns, *hyperactivity of fire due to yin deficiency* and *heart-kidney noninteraction*, whereas fine pulse was also found in two excess patterns, *liver-qi stagnation transforming into fire* and *liver fire flaming upward*. There was also slight difference in pulse feature among the deficiency patterns (Table 2).

### 3.5. Insomnia Symptom Checklist for TCM Diagnostic Purpose

The symptom checklist took into consideration of the common and less common symptoms of the top 10 TCM patterns diagnosed in patients with insomnia (refer to the Methods section). It consisted of 92 items, including 13 sleep-related symptoms, 61 non-sleep-related symptoms, 11 tongue features, and seven pulse features (Table 3). Most of the symptoms included in the checklist were derived from the reviewed studies, with the exception of head distension, abdominal distension, dry tongue, and strong pulse, which were only listed in TCM textbooks [16–19].

## 4. Discussion

This is the first systematic review examining both English and Chinese literatures on the classification of insomnia using the TCM diagnostic system. We conducted an extensive review of 103 articles involving 9499 subjects to derive the common TCM patterns in the diagnosis of insomnia and the clinical features of the TCM patterns. The top 3 TCM patterns *deficiency of both the heart and spleen*, *hyperactivity of fire due to yin deficiency*, and *liver-qi stagnation transforming into fire* covered slightly more than half of the TCM patterns diagnosed in subjects with insomnia. Five of the 10 most common TCM patterns found in our review, namely *deficiency of both the heart and spleen*, *hyperactivity of fire due to yin deficiency*, *liver-qi stagnation transforming into fire*,* internal disturbance of phlegm-heat*, and *qi deficiency of the heart and gallbladder, *were listed in standard TCM textbooks in China [16–18].

We found that the terminology relating to sleep-related symptoms in the TCM classification was much more detailed than those used in the Western diagnostic systems. The insomnia symptoms mentioned in the *Diagnostic and Statistical Manual of Mental Disorders Fourth Edition *[23] and the *WHO International Classification of Diseases 10th Edition* [23] include difficulty falling asleep, difficulty maintaining sleep, nonrefreshing sleep, and nonrestorative sleep. Although excessive dreaming, awakening with a start and restless sleep were common complaints in individuals with insomnia, they were not utilized in the Western diagnostic systems. Half asleep, going to sleep late at night, insomnia with vexation, and difficulty falling asleep with vexation were seldom mentioned in the Western literature. At present, no scientific investigation on these individual sleep symptoms has been performed; hence future studies are needed to determine their clinical significance.

Somatic symptoms are overrepresented in the TCM diagnostic system, whereas psychological symptoms are rarely mentioned. The finding is in line with the fact that Chinese patients use more somatic words to talk about emotions than Western people [24]. We found 92 different non-sleep-related signs and symptoms that were associated with the top 10 TCM patterns for insomnia. These signs and symptoms appear to reflect the imbalance or malfunctioning of various body systems, which can be causes or consequences of insomnia or both. For example, reddish eyes and reddened complexion found in *liver-qi stagnation transforming into fire* may indicate sympatho-excitation [25], whereas reduction in luster complexion seen in *deficiency of both the heart and spleen* may suggest the opposite. Supposedly, the TCM classification system utilizes somatic symptoms and tongue and pulse features to discern differences in etiology and pathogenesis of insomnia and also emphasizes variation in body constitution, whereas the Western system focuses mainly on the etiology of insomnia and puts less emphasis on the pathogenesis and body constitution.

It is worthwhile to note that the TCM patterns commonly found in individuals with insomnia are not unique to insomnia. For this reason, it is rather common to find in TCM that different diseases are treated with the same formula or the same set of acupoints, when the underlying pattern is similar. For example, Gui Pi Tang is used for *deficiency of both the heart and spleen *in insomnia and in dizziness [15]. It is believed that this treatment approach is important for eradicating the underlying cause of diseases [16].

We understand that concrete evidence concerning the value of the TCM diagnostic system in the treatment of insomnia is still unavailable. The usefulness of the large number of non-sleep-related symptoms and the interrater reliability in TCM pattern differentiation are uncertain. A previous study has commented that poor diagnostic reliability can generally be traced to two different sources of uncontrolled variability [26]. The first is information variance, which occurs in the information-gathering process when different levels and types of data are collected about an individual by different interviewers. The other source of variability, criterion variance refers to the use of different sets of rules for classification purpose by different practitioners. Our study showed that the present TCM diagnostic system was subjected to the two sources of variability. There was a lack of standardization in terminology in the current TCM literature. A total of 51 Chinese terms relating to sleep-related symptoms were found, and many similar terms were used to describe non-sleep-related, tongue, and pulse features. The diagnostic criteria used for TCM diagnosis was different among TCM practitioners. It is possible that different researchers may choose the diagnostic criteria at their discretion based on their training and experiences. We consider that; unless a similar set of data is collected, standardized terminology is used, and same rule is applied it is difficult for practitioners to agree on TCM diagnosis.

The symptom checklist derived from our systematic review may reduce the information variance in the TCM diagnosis for insomnia and can be developed into a standardized tool to assess the presence and severity of the symptoms and signs in patients with insomnia. Consistency in symptom recognition between practitioners can be examined. The data can be analyzed using statistical methods such as hierarchical latent class modeling to examine the validity of TCM pattern differentiation [27]. We believe this is an important step in the scientific research of TCM treatment for insomnia.

There are strengths as well as methodologic limitations of the study. Our data were generated from a systematic review of TCM diagnosis in more than 100 articles involving almost 10000 subjects with insomnia, which provided less biased results than those derived from TCM experts. We employed broad inclusion criteria with no specification for the type of study and study quality. This approach could improve generalisability of our findings; however, the quality of data and reliability of the TCM diagnosis were uncertain. The major limitation was that the symptoms and signs of the TCM patterns were based on the description in the studies. It was uncertain whether the clinical features were established by face-to-face interview or based on the practitioners' educational background and clinical experience. In addition, there were insufficient data in the original papers to determine the pathognomonic features and the exclusion criteria that had been used for classification, especially regarding the relationships between TCM patterns and the non-sleep-related symptoms and tongue and pulse features. For example, fine pulse was expected in both *deficiency of both the heart and spleen* and* hyperactivity of fire due to yin deficiency*, but the presence of red tongue would suggest the later TCM pattern. But such exclusion criteria was not stated in the included papers.

## 5. Conclusion

Despite the limitations, the present study, for the first time, systematically and comprehensively summarized important data on the TCM diagnosis of patients with insomnia. We believed that while the TCM classification system had the potential to refine treatment by identifying subtle differences in etiology, pathogenesis, and body constitution, a lack of standardization in terminology and consensus on diagnostic criteria are major barriers. The insomnia symptom checklist derived from our study could be seen as a way of controlling information variance and should be used for future reliability and validity studies.

## Figures and Tables

**Table 1:** The 10 most common TCM patterns for insomnia.

TCM pattern	Chinese name	Subjects with insomnia (*N* = 9499)
		Number of subjects (%)
*Deficiency of both the heart and spleen*	*心脾* *兩虛*	2378 (25.0)
*Hyperactivity of fire due to yin deficiency*	*陰虛* *火旺*	1622 (17.1)
*Liver-qi stagnation transforming into fire*	*肝鬱* *化火*	921 (9.7)
*Heart-kidney noninteraction*	*心腎* *不交*	767 (8.1)
*Qi deficiency of the heart and gallbladder*	*心膽* *氣虛*	544 (5.7)
*Internal disturbance of phlegm-heat*	*痰熱* *內擾*	466 (4.9)
*Liver fire flaming upward*	*肝火* *上擾*	285 (3.0)
*Heart deficiency with timidity*	*心虛* *膽怯*	202 (2.1)
*Stomach disharmony*	*胃腑* *不和*	120 (1.3)
*Stomach qi disharmony*	*胃氣* *不和*	44 (0.5)

**Table 2:** Clinical features of the 10 most common TCM patterns for insomnia.

TCM patterns	Sleep-related symptoms	Non-sleep-related symptoms and signs	Tongue features	Pulse features
Excess patterns				
*Liver-qi stagnation transforming into fire *	**Insomnia**, difficulty falling asleep, excessive dreaming	**Vexation, irritability, bitter taste, constipation, reddish eyes, yellow urine**, headache, dizziness, hypochondriac pain, impatience, reddened complexion, thirst, poor appetite, oppression in the chest, tinnitus, hypochondriac distension, favour of drinking, reddish urine, pain in the chest and hypochondrium, frequent sighing	**Red tongue with yellow coating**	**Rapid and string-like pulse**, fine pulse
*Internal disturbance of phlegm-heat *	Insomnia, restless sleep	**Dizziness, vexation, bitter taste, profuse sputum, oppression in the chest, gastric stuffiness**, heavy headedness, acid regurgitation, poor appetite, belching, headache, nausea	**Red tongue with yellow and slimy coating**	**Slippery and rapid pulse**
*Liver fire flaming* *upward *	**Insomnia**, difficulty falling asleep	**Vexation, bitter taste, dry mouth, reddish eyes, tinnitus, irritability**, constipation, dizziness, dizziness with headache, dry throat, nocturnal emission, feverish sensations in the palms and soles, hypochondriac pain, impatience, reddened complexion, night sweating, palpitation and restless, aphthous stomatitis, backache, poor memory, yellow urine	**Thin coating**, **yellow coating**, red in the tip of tongue, red tongue, scanty coating, no coating	**Rapid**, string-like pulse, fine pulse
*Stomach disharmony *	Difficulty falling asleep, excessive dreaming, difficulty staying asleep, insomnia with vexation, restless sleep, unrefreshing sleep, insomnia, shallow sleep	**Vexation**,** belching**,** dizziness**, dry mouth, dry throat, feverish sensations in the palms, soles, and chest, night sweating, gastric stuffiness, stuffiness and pain in stomach and abdomen, sore knees, backache, hot flashes, constipation, flusteredness, poor appetite, oppression in the chest, stuffiness in stomach and abdomen, sloppy stool, tinnitus	**Slimy coating**, red tongue, scanty coating, thick coating, white coating, yellow coating	**Slippery pulse**, fine pulse, rapid pulse, string-like pulse, weak pulse
*Stomach qi disharmony *	∗	**Abdominal distention**,** belching**	∗	∗
Deficiency patterns				
*Deficiency of both the* *heart and spleen *	**Excessive dreaming, difficulty staying asleep**, difficulty falling asleep, insomnia, half asleep	**Palpitation, lassitude, reduction in luster complexion, poor memory, dizziness, fatigue**, tasteless, weary limbs, poor appetite, sloppy stool	**Pale tongue with thin coating**, white thin coating	**Fine and weak pulse**
*Hyperactivity of fire due* *to yin deficiency *	**Insomnia**, difficulty staying asleep, insomnia with vexation, excessive dreaming, difficulty falling asleep	**Tinnitus, palpitation, poor memory, dizziness, feverish sensations in the palms, soles and chest, dry mouth, backache, vexation**, nocturnal emission, acid regurgitation, sore knees, sweating, dry throat, seminal emission, poor appetite, bitter taste, hot flashes, reddened cheeks	**Red tongue**, scanty coating, slimy coating, white coating, yellow coating	**Fine and rapid pulse**, slippery pulse
*Qi deficiency of the* *heart and gallbladder *	**Insomnia, excessive dreaming, frequent awakening with a start**, difficulty falling asleep, difficulty falling asleep alone, difficulty staying asleep	**Palpitation, fatigue, susceptibility to fright, dyspnea**, pale and large amount of urine, vexation in sitting and lying down, thoughtful	**Pale tongue**, thin coating	**Fine and string-like pulse**
*Heart-kidney noninteraction *	Insomnia, excessive dreaming, difficulty falling asleep, difficulty falling asleep with vexation, insomnia with vexation, difficulty staying asleep, restless sleep	**Backache, dizziness, tinnitus**, palpitation, vexation, feverish sensations in the palms, soles, and chest, seminal emission, night sweating, sore knees, dry mouth, susceptibility to fright, aphthous stomatitis, cold extremities, fright palpitation, irritability, reddened complexion, reddish eyes, poor memory, dry throat, hot flashes, impatience, nocturnal emission, spermatorrhea	**Red tongue**, scanty coating, thin coating, pale tongue, yellow coating, red in the tip of the tongue	**Fine and rapid pulse**, string-like pulse, sunken pulse, weak pulse
*Heart deficiency with timidity *	**Excessive dreaming**, **sleeping late at night**, frequent awakening with a start	**Palpitation**, **susceptibility** to fright, dyspnea, oppression in the chest, gastric stuffiness	**Pale tongue**,** thin coating**, white coating	**Fine and string-like pulse**, weak pulse

Symptoms mentioned in more than 50% of the studies that described the TCM pattern are bolded.

*No study provided information regarding sleep-related symptoms and tongue and pulse features of *stomach qi disharmony*.

**Table 3:** Insomnia symptom checklist for TCM diagnostic purpose.

Sleep-related symptoms (*n* = 13): Difficulty falling asleep, difficulty falling asleep alone, difficulty falling asleep with vexation, difficulty staying asleep, excessive dreaming, frequent awakening with a start, half asleep, insomnia, insomnia with vexation, restless sleep, shallow sleep, sleeping late at night, unrefreshing sleep.	

Non-sleep-related symptoms (*n* = 61): Abdominal distention, acid regurgitation, aphthous stomatitis, backache, belching, bitter taste, cold extremities, constipation, dizziness, dizziness with headache, dry mouth, dry throat, dyspnea, fatigue, favour of drinking, feverish sensations in the palms, soles, and chest, flusteredness, frequent sighing, fright palpitation, gastric stuffiness, headache, **head distension**, heavy headedness, hot flashes, hypochondriac distension, hypochondriac pain, impatience, irritability, lassitude, **menstrual disturbance**, nausea, night sweating, nocturnal emission, **oliguria**, oppression in the chest, pain in the chest and hypochondrium, pale and large amount of urine, palpitation, poor appetite, poor memory, profuse sputum, reddened cheeks, reddened complexion, reddish eyes, reddish urine, reduction in luster complexion or lusterless complexion, seminal emission, sloppy stool, sore knees, spermatorrhea, stuffiness in stomach and abdomen, stuffiness and pain in stomach and abdomen, susceptibility to fright, sweating, tasteless, thirst, thoughtful, tinnitus, vexation, vexation in sitting and lying down, weary limbs, yellow urine.	

Tongue features (*n* = 11): **Dry tongue**, no coating, pale tongue, red in the tip of tongue, red tongue, scanty coating, slimy coating, thick coating, thin coating, white coating, yellow coating.	

Pulse features (*n* = 7): Fine pulse, rapid pulse, slippery pulse, string-like pulse, sunken pulse, **strong pulse**, weak pulse.	

Symptoms only listed in standard TCM textbooks but not in the reviewed studies are bolded.
